# Biomarker analysis of Morquio syndrome: identification of disease state and drug responsive markers

**DOI:** 10.1186/1750-1172-6-84

**Published:** 2011-12-16

**Authors:** Lisa Martell, Kelly Lau, Miranda Mei, Vicki Burnett, Celeste Decker, Erik D Foehr

**Affiliations:** 1 BioMarin Pharmaceutical Inc. Novato, CA USA; 2 SciWit Inc. Henderson, NV USA

## Abstract

**Background:**

This study was conducted to identify potential biomarkers that could be used to evaluate disease progression and monitor responses to enzyme replacement therapy (ERT) in patients with mucopolysaccharidosis (MPS) IVA.

**Methods:**

Levels of 88 candidate biomarkers were compared in plasma samples from 50 healthy controls and 78 MPSIVA patients not receiving ERT to test for significant correlations to the presence of MPSIVA. MPSIVA samples were also tested for correlations between candidate biomarkers and age, endurance, or urinary keratin sulfate (KS) levels. Then, levels of the same 88 analytes were followed over 36 weeks in 20 MPSIVA patients receiving ERT to test for significant correlations related to ERT, age, or endurance.

**Results:**

Nineteen candidate biomarkers were significantly different between MPSIVA and unaffected individuals. Of these, five also changed significantly in response to ERT: alpha-1-antitrypsin, eotaxin, lipoprotein(a), matrix metalloprotein (MMP)-2, and serum amyloid P. Three of these were significantly lower in MPSIVA individuals versus unaffected controls and were increased during ERT: alpha-1-antitrypsin, lipoprotein(a), and serum amyloid P.

**Conclusions:**

Candidate biomarkers alpha-1-antitrypsin, lipoprotein(a), and serum amyloid P may be suitable markers, in addition to urinary KS, to follow the response to ERT in MPSIVA patients.

## Background

Mucopolysaccharidosis (MPS) IVA (OMIM #253000), also known as Morquio A syndrome, is an autosomal recessive lysosomal storage disorder caused by deficient activity of N-acetylgalactosamine-6-sulfatase (GALNS) [1]. GALNS catalyzes the degradation of the glycosaminoglycans (GAGs), KS and chondroitin-6-sulfate (CS). In individuals affected with MPSIVA, KS and CS accumulate in the tissues, causing skeletal dysplasia, coarse facial features, restricted growth and short stature, joint hypermobility, valvular heart disease, pulmonary disease, obstructive sleep apnea, hepatomegaly, corneal clouding, hearing loss, and poorly formed teeth [1]. Because more than 150 mutations in the gene encoding GALNS have been identified, there is considerable clinical heterogeneity ranging from mild to severe based on the residual GALNS activity [1,2]. The International Morquio Registry and the Morquio Clinical Assessment Program (MorCAP, also known as MOR-001) found considerable morbitity and mortality in MPSIVA individuals, including frequent surgical procedures, limited mobility, poor endurance and pulmonary function, and death in the second or third decade of life for patients with a severe phenotype (about 68% of MPSIVA patients) [1,3]. Urinary GAG testing is used to screen for MPSIVA, with enzyme activity measurement in blood or fibroblasts to confirm diagnosis [1,2]. The incidence of MPSIVA ranges from 1 in 40,000 to 1 in 450,000 live births [1,2].

Currently there is no treatment for MPSIVA, only supportive measures such as surgeries for skeletal dysplasia, oxygen for poor lung function, and antibiotics for lung infections [1,2]. However, ERT with recombinant human (rh) GALNS (BMN110) is under clinical development, ERT provides a supply of the deficient enzyme and is available for MPS I, MPS II, and MPS VI [4].

In a mouse model of MPSIVA [5]. rhGALNS markedly decreased storage in visceral organs, heart valves, ligaments, connective tissues, and brain [6]. ERT (rhGALNS, BMN110) for MPSIVA is evaluated in a Phase I/II human trial. The clinical trial is a multicenter, open-label, dose-escalation study to evaluate safety, tolerability, and efficacy of BMN 110 in patients with MPS IVA (clinical trial #NCT00884949) between 5 and 18 years of age [7]. A global double blind, randomized, placebo-controlled Phase III study of BMN 110 in MPSIVA was initiated in 2011 [8].

With ERT in development for MPS IVA, identification of biomarkers to evaluate disease progression and response to treatment becomes more important. To date, KS has been identified as an important biomarker for MPS IVA [9] as it accumulates in the tissues, particularly the cartilage, cornea, and heart valves, in MPS IVA patients [10]. KS storage in the cartilage causes cartilage disruption, which contributes to increased circulating levels of KS in MPS IVA patients relative to healthy individuals. KS can be quantified in urine and plasma and may be helpful for screening and for following the clinical course and efficacy of treatment [9,11]. KS levels in urine and plasma have been shown to vary with age (with plasma levels generally peaking between 5 to 10 years of age and urine levels peaking between 1 and 5 years of age) and increase with clinical severity of MPS IVA [12].

While KS is an important and treatment-related biomarker to track MPSIVA disease progression and response to ERT, it is not known whether there are additional biomarkers associated with MPS IVA and their relationship to KS levels, disease severity, disease progression and response to ERT. The goal of the current study is to identify additional candidate biomarkers that support evaluations of disease progression and monitoring response to ERT and examine their relationships with levels of KS.

## Methods

### Analytes

To identify and evaluate potential biomarkers for MPS IVA, this study examined 88 different analytes from plasma samples obtained from 50 healthy adult control subjects, 78 MPS IVA subjects not receiving ERT from the MOR-001 study, and 20 subjects receiving ERT with BMN 110 from the MOR-002 study. The analytes for this study were from the Human Multi-Analyte Profile (MAP) v1.6 from Rules Based Medicine (Austin TX). This is an 88-biomarker MAP providing quantitative multiplexed assays covering different biological pathways. Biomarkers included in this MAP were selected to provide a comprehensive understanding of complex drug and disease effects. The analytes on the Human MAP v1.6 are a pre-set panel and were not custom selected for this particular disease study. Analysis of samples was performed using the Luminex platform.

### MOR-001

The MOR-001 study is a multicenter, multinational, longitudinal clinical assessment of patients with MPS IVA not receiving ERT. This longitudinal study aims to catalogue the spectrum of symptoms and biochemical abnormalities of subjects with MPS IVA. There have been 129 subjects enrolled through 2009 [3]. Analytes were profiled and quantified from plasma samples obtained from the initial visit of the first 78 of MPS IVA subjects and compared with the samples from 50 controls subjects (61% female, female age range 21-70, mean female age 45; 39% male, male age range 24-71, mean male age 47). Analyte profiles obtained in the MPSIVA group were then sub-analyzed to determine if any significant correlations existed to subject age, performance in an endurance test (6 minute walk test; 6 MWT), or levels of urinary KS (uKS).

### MOR-002

The MOR-002 study is a multicenter, open-label, dose-escalation study to evaluate the safety, tolerability, and efficacy of BMN 110 in patients with MPS IVA between 5 and 18 years of age. Twenty subjects enrolled in the study, and 18 subjects completed 36 weeks of therapy with weekly 4 to 5 hours intravenous infusions of BMN 110. The study used 3 consecutive 12-week dosing intervals (the dose escalation phase), using the following regimen:

Weeks 1012: 0.1 mg/kg/week

Weeks 13-24: 1.0 mg/kg/week

Weeks 25-36: 2.0 mg/kg/week

Subjects completing the 36-week dose-escalation period had the option to continue drug treatment for an additional 36 weeks. Subjects continuing treatment after the dose-escalation period received weekly 4 to 5 hour intravenous (IV) infusions of BMN 110 at a dose of 1.0 mg/kg/week. Clinical outcomes included measures of endurance (6 MWT), 3 minute stair climb test (3 MSCT) and pulmonary function (forced vital capacity [FVC]), examined at baseline and weeks 6, 12, 18, 24, 30, 36, 48, 60, and 72 [7].

The same 88 analytes studied in MOR-001 patients were profiled longitudinally in 20 MOR-002 study subjects at baseline, 12, 24, and 36 weeks post-ERT during the dose escalating phase. Analyte profiles were then sub-analyzed to determine if any significant correlations existed to subject age or performance in the 6 MWT.

Both MOR-001 and MOR-002 were approved by an Institutional Review Board and informed consent was obtained from all human subjects.

### Statistical analysis

T-tests were performed to check for significant differences in analytes between MPS IVA patients and unaffected control subjects (for MOR-001), between older (over 9.5 years) and younger (9.5 years or less) MPS IVA subjects, between MPS IVA subjects with high (> 24.6 μg/mg creatinine) and low (≤ 24.6 μg/mg creatinine) uKS, and between MPS IVA subjects with high (> 235 meters) and low (≤ 235 meters) 6 MWT scores. The specific high and low cut-off points for age, uKS and 6 MWT scores were selected to keep the groups statistically balanced. T-tests were also used to compare baseline and the different time points of ERT (baseline vs. week 12, baseline vs. week 24, and baseline vs. week 36, week 12 vs. week 24, week 12 vs. week 36, week 24 vs. week 36). T-tests were performed using the unpooled variance method. For patients on ERT, analysis of variance (ANOVA) was carried out across all time points, over all samples and all analytes on the normalized data, and subsequent Tukey results (a statistical test performed after ANOVA to perform a pairwise comparison of the means) are presented.

## Results

### Demographics

Table 1 summarizes the demographic information for MPS IVA subjects from MOR-001 and MOR-002 participating in this biomarker study. The 78 subjects selected from the MOR-001 study for this analysis are demographically matched to the overall MOR-001 study population.

**Table 1:** Subject demographics

	Demographic Variables	MOR-001 (N = 78)	MOR-002 (N = 20)
Age (years)	Mean (SD)	13.47 (12.41)	7.95 (2.892)
	Median	9.5	7.5
	Min, Max	1.0, 65.0	4.0, 16.0

Ethnicity	Hispanic or Latino	3 (3.8%)	0 (0%)
	Not Hispanic or Latino	75 (96.2%)	20 (100%)

Race	White	41 (52.6%)	9 (45.0%)
	Asian	24 (30.8%)	9 (45.0%)
	Black or African American	0 (0%)	0 (0%)
	Other	13 (16.7%)	2 (10%)

Gender	Female	42 (53.8%)	8 (40.0%)
	Male	36 (46.2%)	12 (60.0%)

### Comparison of MPS IVA subjects (did not receive ERT; MOR-001) to unaffected control group

Nineteen analytes were significantly different between these two groups at the p < 0.05 level (see Table 2). The pattern of values for these nineteen analytes in MPS IVA patients was different from the unaffected controls in almost all subject samples. Three analytes were significantly higher in MPS IVA subjects versus unaffected controls, and the other 16 were significantly lower in MPS IVA subjects compared to controls.

**Table 2:** Analytes that differ significantly between MPS IVA and unaffected control subjects

Analyte	p-value
**Significantly higher in MPSIVA patients versus unaffected controls**	

MDC (macrophage-derived chemokine	0.047
MIP-1 alpha (macrophage inflammatory protein-1 alpha)*	0.014
MMP-2 (matrix metalloproteinase-2)*†	0.003

**Significantly lower in MPSIVA patients versus unaffected controls**	

Alpha-1-antitrypsin	0.006
Alpha-fetoprotein	0.001
C-reactive protein	0.000
EN-RAGE (extracellular newly-identified receptor for advanced glycosylation end products)	0.000
Eotoxin	0.001
Erythropoietin	0.000
Factor VII	0.028
Ferritin	0.019
IFN-gamma (interferon-gamma)	0.001
IL-RA (interleukin-1 receptor antagonist)	0.000
Leptin*	0.000
Lipoprotein (a)*	0.000
MCP-1 (monocyte chemotactic protein 1)	0.009
Myeloperoxidase	0.036
Prostatic acid phosphatase	0.000
Serum amyloid P*	0.006

* These analytes were also significantly different between the high and low uKS groups of MPS IVA patients (see table 5).

† This analyte was also significantly different between the older and younger age groups of MPS IVA patients (see table 3).

Note: The following analytes were also tested but not found to be statistically significantly different between MPS IVA and unaffected individuals: adiponectin, alpha-2-macroglobulin, apolipoprotein A-I, apolipoprotein C-III, apolipoprotein H, apolipoprotein (a), beta-2-microglobulin, brain-derived neurotrophic factor, calcitonin, cancer antigen 125, cancer antigen 19-9, carcinoembryonic antigen, CD40 antigen, CD40 ligand, complement C3, creatine kinase-MB, endothelin-1, epidermal growth factor, epithelial-derived neutrophil-activating protein 78, fatty acid-binding protein (heart), fibrinogen, fibroblast growth factor basic, granulocyte colony-stimulating factor, granulocyte-macrophage colony-stimulating factor, growth hormone, haptoglobulin, immunoglobulin A, immunoglobulin E, immunoglobulin M, insulin, insulin-like growth factor I, intercellular adhesion molecule 1, interleukin-1 alpha, interleukin-1 beta, interleukin-2, interleukin-3, interleukin-4, interleukin-5, interleukin-6, interleukin-7, interleukin-8, interleukin-10, interleukin-12 subunit p40, interleukin-12 subunit p70, interleukin-13, interleukin-15, interleukin-16, lymphotactin, macrophage inflammatory protein 1-beta, matrix metalloproteinase-3, matrix metalloproteinase-9, myoglobulin, plasminogen activator inhibitor 1, pregnancy-associated plasma protein A, prostate-specific antigen (free), RANTES/CCL5 (regulated upon activation, normal T-cell expressed and secreted), serum glutamic oxaloacetic transaminase, sex hormone-binding globulin, stem cell factor, thrombopoietin, thyroid-stimulating hormone, thyroxine-binding globulin, tissue factor, tissue inhibitor of metalloproteinase 1, tumor necrosis factor alpha, tumor necrosis factor beta, tumor necrosis factor receptor-like 2, vascular cell adhesion molecule-1, vascular endothelial growth factor, and von Willebrand factor.

### Analyte correlations in MPS IVA subjects not receiving ERT

#### Correlation with age

Table 3 lists the 11 analytes that were significantly different between the high (> 9.5 years) and low (≤ 9.5 years) age groups for MPS IVA subjects. Of these, 3 were significantly higher in the older patients, and 8 were significantly higher in the younger patients. Six of the 11 analytes were also significantly different between the high and low uKS groups. MMP-2 was also significantly different between MPS IVA subjects and unaffected controls (see Table 2).

**Table 3:** Analytes significantly correlated with age in MPS IVA subjects

Analyte	p-value
**Significantly higher in older patients (> 9.5 years) versus younger patients**	

IGF-1 (insulin-like growth factor-1)	0.010
IgA (immunoglobulin A)*	0.002
IgM (immunoglobulin M)*	0.004

**Significantly higher in younger patients (< 9.5 years) versus older patients**	

Calcitonin	0.015
G-CSF (granulocyte colony-stimulating factor)	0.044
Growth hormone*	0.003
ICAM-1 (intracellular adhesion molecule-1)*	0.000
MMP-2 (matrix metalloproteinase-2)*‡	0.019
TNF-alpha (tumor necrosis factor alpha)*	0.000
TSH (thyroid stimulating hormone)	0.023
VCAM-1 (vascular cell adhesion molecule-1)*	0.009

* These analytes were also significantly different between high and low uKS groups (see Table 5).

‡This analyte was also significantly different between MPS IVA patients and unaffected controls (see Table 2).

#### Correlations with 6 MWT score

Table 4 lists the 7 analytes that were significantly different between the MPS IVA subjects with high versus low 6 MWT scores (> 235 meters), and the other three were higher in patients with low 6 MWT scores (≤ 235). The analyte insulin was also significantly different between high and low uKS groups.

**Table 4:** Analytes significantly correlated with 6 MWT performances in MPS IVA subjects

Analyte	p-value
**Significantly higher in patients with high 6MWT score (> 235) versus lower scores**	

Apolipoprotein A1	0.011
Apolipoprotein CIII	0.001
Insulin*	0.004
Myoglobin	0.029

**Significantly higher in patients with low 6MWT score (< 235) versus higher scores**	

Adiponectin	0.013
Alpha-2 macroglobulin	0.002
CD40 ligand (cluster of differentiation 40 ligand)	0.034

*This analyte was also significantly different between high and low uKS groups (see Table 5).

#### Correlations with uKS levels

Table 5 lists the 16 analytes that were significantly different between the MPS IVA subjects with high versus low urinary KS. Nine of these analytes were significantly higher in subjects with high uKS (> 24.6 μg/mg creatinine), and seven were significantly higher in subjects with low uKS (≤ 24.6 μg/mg creatinine).

**Table 5:** Analytes significantly correlated with uKS levels in MPS IVA subjects

Analyte	p-value
**Significantly higher with high uKS (> 24.6 g/mg creatinine) versus lower levels**	

Alpha-2 macroglobulin†	0.029
Growth hormone†	0.003
ICAM-1 (intracellular adhesion molecule-1)†	0.007
MIP-1 alpha (macrophage inflammatory protein-1 alpha)‡	0.022
MMP-2 (matrix metalloproteinase-2)‡	0.024
SHBG (sex hormone-binding globulin)	0.029
TNF-alpha (tumor necrosis factor alpha)†	0.005
Thyroxine binding globulin	0.000
VCAM-1 (vascular cell adhesion molecule-1)†	0.003

**Significantly higher with low uKS (< 24.6 μg/mg creatinine) versus higher levels**	

Brain-derived neurotrophic factor	0.045
IgA (immunoglobulin A)†	0.008
IgM (immunoglobulin M)†	0.005
Insulin§	0.011
Leptin‡	0.010
Lipoprotein (a)‡	0.032
Serum amyloid P‡	0.005

† These analytes were also significantly different between the high and low age groups.

‡ These analytes were also significantly different between MPS IVA patients and unaffected controls.

§ This analyte was also significantly different between the high and low 6MWT groups.

### Analyte correlations in MPS IVA subjects post-ERT

Patients with MPS IVA treated with ERT at escalating doses for 36 weeks demonstrated an initial increase in pro-inflammatory and acute phase proteins, followed by a decreased and subsequent modulation of the immune response. Two subjects terminated the study before 12 weeks; neither subject was included in the 12 week time point group.

#### Longitudinal/dose escalating analysis of analyte correlations post-ERT in MPS IVA

Using ANOVA to compare all analytes across all time points (baseline vs. 12, 24, and 36 weeks of ERT), 15 analytes had highly significant changes (p < 0.01) compared to baseline (pre-treatment)-see Table 6. Of these, four analytes were also significantly different between MPS IVA patients not receiving ERT and unaffected controls (see Table 6). These analytes may be candidate biomarkers for monitoring ERT response in patients with MPS IVA.

**Table 6:** Analytes that changed significantly during ERT

Analyte	Effect of ERT on analyte	p-value
**Analytes that increased during ERT**		

Alpha-1-antitrypsin ‡¥	Increase	0.001
Apolipoprotein H	Increase	0.021
Complement 3 ¥	Increase	0.000
Endothelin-1 ¥	Increase	0.000
Fibrinogen	Increase	0.039
IgA (immunoglobulin A)	Increase	0.044
IgM (immunoglobulin M) ¥	Increase	0.000
IGF-1 (insulin-like growth factor 1)	Increase	0.022
Lipoprotein (a) ‡	Increase	0.037
Serum amyloid P ‡¥	Increase	0.001
von Willebrand factor ¥	Increase	0.003

**Analytes that decreased during ERT**		

CD40 ¥	Decrease	0.002
Eotaxin ‡	Decrease	0.020
IL-3 (interleukin-3) ¥	Decrease	0.000
IL-13 (interleukin-13)	Decrease	0.015

‡ These analytes were also significantly lower in MPS IVA patients compared to unaffected controls not receiving ERT.

¥These analytes were statistically significant at the p < 0.01 level for ANOVA comparing analytes across all time points.

The week 12 measures were most different from week 24 and 36, with the week 24 measures more similar to the week 36 measures. Complement 3 is highly significant across all comparisons (except baseline vs. week 12). Alpha-1-antitrypsin is also highly significant across most comparisons (except baseline to week 12 and week 24 to week 36). The other tope analytes are: endothelin-1, IgM, IL-3, IL-13, serum amyloid P, and CD40. Of these 8 top analytes, alpha-1-antitrypsin and serum amyloid P are particularly promising as candidate biomarkers as they were significantly different between MPS IVA and unaffected control subjects not receiving ERT and serum amyloid P also correlated with uKS levels. IgM is also of interest as it was significantly higher in patients with low uKS levels and increased during ERT.

#### Inflammation-related analytes

**Figure 1:**
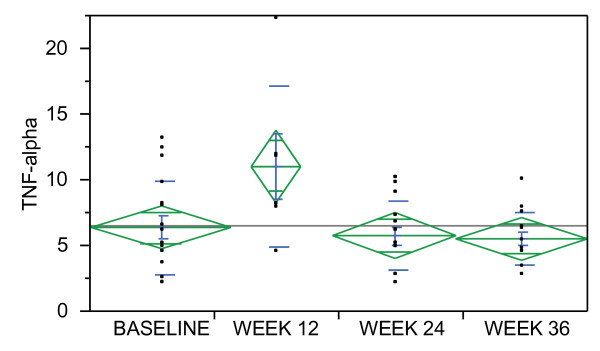
**The green diamonds represent the sample mean and the 95% confidence interval**. The center line across each diamond corresponds to the group mean. The vertical span of each diamond represents the 95% confidence interval for each group. The width and height of the diamonds is proportional to the sample size at each time point with narrow and tall diamonds indicating fewer data points. The blue lines found within the green diamonds are mean error bars corresponding to the mean of each group with error bars one standard error above and below each group mean. The blue lines outside the diamonds represent one standard deviation above and below the group mean. The black squares represent individual data points.

An initial increase in pro-inflammatory analytes (such as TNF-alpha) and acute phase proteins were followed by a decrease or subsequent modulation of the immune response (see Table 7 for longitudinal analysis and Figure 1).

**Table 7:** Longitudinal presentation of analyte data post-ERT (baseline, 12, 24, and 36 weeks).

Comparisons versus baseline			
	Week 12 (p < 0.01)*	**Week 24 (p < 0.01)**	**Week 36 (p < 0.001)**

**Increased over baseline**	Apoliprotein CIII	Alpha-1-antitrypsin	Alpha-1-antitrypsin
	MCP-1	Apolipoprotein CIII	Apolipoprotein CIII
	MMP-2	Complement 3	Complement 3
	Thrombopoietin	Endothelin-1	MMP-2
		Fibrinogen	IgM
		MCP-1	Fibrinogen
		MIP-1 alpha	
		MMP-2	

**Decreased over baseline**	Alpha-2 macroglobulin	IL-4	CD40
	SGOT	IL-13	SGOT
	VCAM-1	IL-1 alpha	Apoliprotein A1
		SGOT	
		VCAM-1	

**Comparison between treatment weeks**			

	**Week 12 vs. 24 (p < 0.05)**	**Week 12 vs. 36 (p < 0.01)**	**Week 24 vs. 36 (p < 0.05)**

**Increased over time**	Alpha-1-antitrypsin	Alpha-1-antitrypsin	Complement 3
	Apolipoprotein H	Apolipoprotein H	
	Complement 3	Complement 3	
	Endothelin-1	Endothelin-1	
	IgM	IGF-1	
	IgA	IgM	
	MIP-1 alpha	Serum amyloid P	
	Serum amyloid P	TNF-alpha	
	von Willebrand Factor		

**Decreased over time**	Alpha-fetoprotein	Apolipoprotein A1	CD40
	Fatty acid binding protein	CD40	
	IL-3	IL-3	
	IL-4		
	IL-7		
	IL-13		
	Thrombopoietin		

*T-tests were performed at a variety of different p-values including p < 0.05, p < 0.01, and p < 0.001. The significance level that gave the best separation between groups is reported.

#### Analyte correlation with age

Table 8 shows the analytes that were significantly different between children aged 5-7 years (younger age group) and those aged 8-10 years (older age group) post-ERT. At week 36 of treatment, the two age groups could be almost completely differentiated using the 6 analytes significant at the p < 0.05 level (see Table 8). Only one of the analytes identified (alpha-1-antitrypsin) was also significantly affected by ERT.

**Table 8:** Analyte correlation with age post-ERT

Analyte response	Week 12	Week 24	Week 36
**Higher in younger patients**	G-CSF	Alpha-1-antitrypsin	Beta-2 microglobulin
			Ferritin
			IL-1 RA
			SGOT
			Stem cell factor

**Higher in older patients**	Apolipoprotein CIII	None	Creatine kinase-MB

#### Age correlation with 6 MWT

Using the eight analytes identified as highly significant between the dose/time points, only alpha-1-antitrypsin correlated with 6 MWT score, and this was a weak negative correlation (R = -0.709) found only at week 12 (but not weeks 24 or 36). None of the other analytes correlated with the 6 MWT, although complement 3 has a strong correlation with IgM and alpha-1-antitrypsin.

## Discussion

This study represents a comprehensive analysis of biomarkers in MPS IVA. Understanding and identifying relevant biomarkers for MPS IVA may help elucidate the biochemical nature of the disease pathology and monitor response to treatment. Of the 88 analytes tested, 19 analytes were able to be differentiated between MPS IVA individuals and unaffected controls. Five of these analytes also changed significantly in response to ERT in the overall ANOVA analysis: alpha-1-antitrypsin, eotaxin, lipoprotein(a), MMP-2, and serum amyloid P. Of these, only 3 were significantly lower in MPS IVA individuals and also increased during ERT: alpha-1-antitrypsin, lipoprotein(a), and serum amyloid P. MMP-2 was significantly higher in MPS IVA individuals but increased with ERT, and exotoxin was significantly lower in MPS IVA individuals but decreased with ERT. Alpha-1-antitrypsin is the only one of these candidate biomarkers that also correlated with the clinical endpoint 6 MWT (at week 12 of treatment).

The magnitude of the number of analyte differences seen between MPS IVA subjects and healthy controls may be explained by the role GAGs such as KS play in cellular signaling, both as free molecules and attached to proteins to form proteoglycans. GAGs may influence cell division, differentiation, signal transduction, adhesion, and response to growth factors. Thus, through cellular signaling, GAGs may affect a variety of biological pathways, including those both related and unrelated to MPS IVA [13,14]. It is important to note that although both the unaffected control group and the MPS IVA subject group covered a large age span (1 to 65 for MPS IVA subjects and 21 to 71 for healthy controls), the unaffected control population had a higher average age than the MPS IVA population, which could theoretically account for some of the observed differences in biomarkers between these two groups. However, most of the biomarkers that were significantly different between these two groups were not significantly correlated with age in MPS IVA patients. As well, age differences between controls and MPS IVA subjects would not have any effect on the other analysis, which compared results within age groups of MPS IVA subjects rather than with controls.

Because uKS has already been identified as an important biomarker for MPS IVA, with high uKS levels being a marker for more severe disease [12], this study also examined correlations between uKS and the candidate biomarkers. Some, but not all of the 9 analytes that were significantly higher in MPS IVA subjects with high uKS changed in response to ERT. The reason for this finding is unknown; perhaps the subset of patients with more severe disease may have required a longer duration of ERT to see a significant change in some analytes, or some analytes may be correlated with pathways that do not respond to ERT. Of the three candidate biomarkers that were significantly lower in MPS IVA individuals and also increased with ERT, two correlated with uKS levels in subjects not receiving ERT. Lipoprotein(a) and serum amyloid P levels were significantly higher in patients with low uKS levels. Further research is needed to determine whether this is due to an association with less severe disease or whether it is related to the fact that KS levels decline with age [12].

Although the role of these biomarkers and their pathophysiology with MPS IVA needs further research, three biomarkers were identified that differentiated MPSIVA from unaffected individuals and correlated with ERT response: alpha-1-antitrypsin, serum amyloid P, and lipoprotein(a). Alpha-1-antitrypsin is an acute phase protein produced by the liver in response to stimuli such as inflammatory mediators. Its primary physiological role is to protect the alveolar tissue of the lungs from proteolytic damage from enzymes such as neutrophil elastase. Deficiency of alpha-1-antitrypsin can produce emphysema, liver cirrhosis, and panniculitis [15]. Therefore, conceivably, the lower levels of alpha-1-antitrypsin seen in MPS IVA patients may add to the lung and liver pathology seen in these patients. The increase in alpha-1-antitrypsin following ERT may be at least partly related to a compensatory response to protect the lungs from damage due to increased proteases (MMP-2) that are clearing the damaged tissue and GAG accumulation. Serum amyloid P is a glycoprotein that forms a major part of serum and plays a role in the acute phase response and complement mediated immunity. It also facilitates the clearance of apoptotic cells [16]. The relationship between serum amyloid P and MPS IVA may be related to inflammation and apoptosis.

Lipoprotein(a) is composed of the protein apolipoprotein(a) linked to Apo-B-100 by a disulfide bond. Elevated lipoprotein(a) levels are associated with an increased risk of atherosclerosis-related events such as cardiovascular disease and stroke. Lipoprotein(a) is believed to be involved in forming atherosclerotic plaques and enhancing platelet interaction [17]. It also correlates with inflammatory activity, as seen in patients with rheumatoid arthritis (RA) [18]. Lipid metabolism is altered in inflammatory states such as RA: levels of atherogenic lipoprotein(a) are elevated and high-density lipoprotein is altered from its usual anti-artherogenic state to become proinflammatory, leading to an increased risk of cardiovascular disease [19]. Alterations in lipid profile are seen even up to 10 years before RA appears [20]. Given the physiological role of lipoprotein(a), it might be expected to contribute to the cardiovascular disease seen in MPS IVA patients. But contrary to expectations, lipoprotein(a) levels were lower in MPS IVA patients compared to unaffected controls. Lipoprotein(a) levels increased with ERT. This may be related to the immune response to ERT shown by the transient increase seen in pro-inflammatory biomarkers and acute phase proteins.

Although MPS disorders are not primarily associated with clinically detectable inflammation, the pathophysiology of bone damage in MPS may involve inflammatory cytokines such as TNF-alpha, interleukin-beta, and substances involved in Toll-like pathways [21]. As well, lysosomal storage of GAGs can impair autophagy, leading to the accumulation of polyubiquitinated proteins and dysfunctional mitochondria, leading to inflammation and apoptosis [22]. This study found higher TNF-alpha levels in patients with high levels of uKS but there was no significant difference in TNF-alpha levels between individuals with MPS IVA and healthy controls. This could be due to the fact that TNF-alpha may play a role in cartilage damage, and patients with higher KS levels have more cartilage damage (since KS is released when cartilage is damaged). TNF-alpha also showed an increase at week 12 of ERT, followed by a decrease to baseline. This may be related to an immune response to ERT, and also has parallels to RA pathology. TNF-alpha is one of the main pro-inflammatory cytokines involved in RA pathology, inducing a complex cascade of inflammatory reactions that lead to joint damage. Research suggests that TNF-alpha plays a role in cartilage damage in RA [23]. Anti-TNF therapy is used to target this cascade to help treat RA and other inflammatory diseases [24]. Both RA and MPS IVA involve damage to the cartilage, so it is not surprising that TNF-alpha would be elevated both in RA patients and in patients with MPS IVA that have high KS levels.

Other markers of inflammation showed mixed results. Two pro-inflammatory markers (MDC and MIP-1 alpha) were higher and one anti-inflammatory marker (IL-1RA) was lower in MPSIVA patients compared with healthy controls. However, many pro-inflammatory markers such as C-reactive protein, eotaxin, IFN-gamma, MCP-1, and serum amyloid P, were actually lower in MPS IVA patients. EN-RAGE was also lower in MPS IVA patients, which would be expected to indicate lower inflammation. It could be that the pathology of MPS IVA involves only specific inflammatory molecules, and further understanding of these pathways will help clarify which markers should be elevated in MPS IVA.

MPSIVA biomarker research is particularly important now that ERT is in development for MPS IVA. Studies in the related lysosomal storage disorders MPS I [25] and MPS VI [26] show that early treatment with ERT may help delay or prevent the onset of MPS symptoms. The current biomarker study found 5 analytes that were significantly higher in younger (aged 5 to 7 years) than in older children (aged 8 to 10 years) after 36 weeks of ERT: beta-2 microglobulin, ferritin, IL-1RA, SGOT, and stem cell factor, one that was significantly lower: creatine kinase-MB. As the development of ERT for MPS IVA progresses, further research will be needed to identify potential biomarkers for younger age groups (less than 5 years of age). Additional research may also help clarify the role that body weight plays in Morquio biomarkers, since patients with Morquio syndrome may become overweight due to mobility issues. In the current study, all subjects receiving ERT had antibodies to rhGALNS by week 12 of treatment. This is supported by the longitudinal biomarker evidence which shows an initial increase in pro-inflammatory biomarkers and acute phase proteins, followed by a decrease in the immune responses. Therefore, some of the changes in the listed biomarkers are likely a result of the anti-rhGALNS immune response rather than indicators of ERT response. It should be noted that a high level of antibody response to ERT has been observed in other lysosomal storage disorders including MPS I, II, and VI. In the majority of cases, available evidence has not demonstrated a correlation between antibody development and changes in safety or efficacy [27].

## Conclusions

This study identified biomarkers that may help elucidate the biochemical nature of the disease pathology and monitor responses to therapy. Biomarkers of particular interest for further research include alpha-1-antitrypsin, lipoprotein(a), and serum amyloid P. While any of these individual analytes may be candidates for biomarkers, all analyses were carried out using sets of analytes; individual analytes and all possible combinations were not tested. Follow-up laboratory tests will be necessary to verify the candidate biomarkers and further explore their role in MPS IVA. Future research may also help evaluate the role of other candidate biomarkers that were not available in the panel used for this study, such as collagen 1 and 2. The Human MAPv1.6 panel of biomarkers did not have a comprehensive set of bone/cartilage specific markers and therefore future studies could focus on biochemical markers of skeletal abnormalities. Abnormal levels of KS and other matrix components may directly impact the inflammatory response. The intracellular and extracellular accumulation of KS and ongoing damage to certain tissues in MPSIVA appear to cause a chronic inflammatory state with an imbalance in many physiological systems. Future cell based or disease model studies could shed light on the role of KS in inflammation.

## Competing interests

The authors declare that they have no competing interests.

## Authors' contributions

LM participated in the design, coordination, and analysis, KL participated in the statistical analysis, MM participated in the coordination of the study. VB performed statistical analysis. CD participated in the design of the study. EF participated in the design of the study and data interpretation. All authors read and approved the final manuscript.

## References

[B1] Montano AM Tomatsu S Gottesman GS Smith M Orii T International Morquio A Registry: Clinical manifestation and natural course of Morquio disease J Inherit Metab Dis 2007 30 165 174 10.1007/s10545-007-0529-7 17347914

[B2] Lankaster BJA Whitehouse M Gargan MF Morquio syndrome Curr Orthop 2006 20 128 131 10.1016/j.cuor.2005.12.003

[B3] Harmatz P Chang M Decker C A multicenter, multinational, longitudinal clinical assessment study of subjects with mucopolysaccharidosis IVA (Morquio syndrome) Poster presented at the Society of the Study of Inborn Errors of Metabolism (SSIEM) 2010 Meeting. Istanbul 2010

[B4] Lim-Melia ER Kronn DF Current enzyme replacement therapy for the treatment of lysosomal storage diseases Pediatr Ann 2009 38 448 455 10.3928/00904481-20090723-09 19725195

[B5] Tomatsu S Orii KO Vogler C Mouse model of N-acetylgalactosamine-6-sulfate sulfatase deficiency (*Galns -/-*) produced by targeted disruption of the gene defective in Morquio A disease Hum Mol Genet 2003 12 3349 3358 10.1093/hmg/ddg366 14583446

[B6] Tomatsu S Montano A Ohashi A Enzyme replacement therapy in a murine model of Morquio A syndrome Hum Mol Genet 2008 17 815 824 18056156 10.1093/hmg/ddm353

[B7] BioMarin Pharmaceuticals Inc. A phase 1/2, multicenter, open-label, dose escalation study to evaluate the safety, tolerability, and efficacy of BMN110 in subjects with mucopolysaccharidosis IVA Clinical study protocol, NCT00884949

[B8] BioMarin Pharmaceuticals Inc. A double-blind study to evaluate the efficacy and safety of BMN110 in patients with mucopolysaccharidosis IVA Clinical study protocol, NCT01275066

[B9] Tomatsu S Montano AM Oguma T Validation of keratin sulfate level in mucopolysaccharidosis type IVA by liquid chromatography-tandem mass spectrometry J Inherit Metab Dis 2010 10.1007/s10545-009-9013-x 20107903

[B10] Dvorak-Ewell M Wendt D Hague C Enzyme replacement in a human model of mucopolysaccharidosis IVA in vitro and its biodistribution in the cartilage of wild type mice PLOS ONE 2010 5 8 e12194 10.1371/journal.pone.0012194 20808938 PMC2922370

[B11] Argento-Martell L Cunico RL Ohh J Furneaux RH Foehr ED Validation of an LC/MS/MS assay for detecting relevant disaccharides from keratan sulfate as a biomarker for Morquio syndrome Bioanalysis 2011 3 1855 1866 10.4155/bio.11.172 21877895

[B12] Tomatsu S Okamura K Taketani T Development and testing of a new screening method for keratan sulfate in mucopolysaccharidosis IVA Pediatr Res 2004 55 592 597 10.1203/01.PDR.0000113767.60140.E9 14711889

[B13] Conrad AH Zhang Y Tasheva ES Conrad GW Proteomic analysis of potential keratan sulfate, chondroitin sulfate A, and hyaluronic acid molecular interactions Invest Ophthamol Vis Sci 2010 51 4500 4515 10.1167/iovs.09-4914 PMC2941170 20375348

[B14] Wilson S Hashamiyan S Clarke L Collagenolytic activity in MPSI contributes to osteoclast and growth plate abnormalities Am J Pathol 2009 175 2053 2062 10.2353/ajpath.2009.090211 19834056 PMC2774069

[B15] Fregonese L Stolk J Hereditary alph-1-antitrypsin deficiency and its clinical consequences Orphanet J Rare Dis 2008 3 16 10.1186/1750-1172-3-16 18565211 PMC2441617

[B16] Yuste J Botto M Bottoms SE Brown JS Serum amyloid P aids complement mediated immunity to *Streptococcus pneumonia* PLOS Patholog 2007 3 e120.doi:10 10.1371/journal.ppat.0030120 PMC1971117 17845072

[B17] Bermudez V Arraiz N Aparicio D Lipoprotein(a): From molecules to therapeutics Am J Ther 2010 17 263 273 10.1097/MJT.0b013e3181e00bf1 20479580

[B18] Choy E Sattar N Interpreting lipid levels in the context of high-grade inflammatory states with a focus on rheumatoid arthritis: a challenge to conventional cardiovascular risk factors Ann Rheum Dis 2009 68 460 469 10.1136/ard.2008.101964 19286905

[B19] Hahn BH Grossman J Ansell BJ Altered lipoprotein metabolism in chronic inflammatory states: proinflammatory high-density lipoprotein and accelerated atherosclerosis in systemic lupus erythematosus and rheumatoid arthritis Arthritis Res Ther 2008 10 213 10.1186/ar2471 18828865 PMC2575639

[B20] Van Halm VP Nielen MM Nurmohamed NT Lipids and inflammation: serial measurements of the lipid profile of blood donors who later developed rheumatoid arthritis Ann Rheum Dis 2007 66 184 188 16760255 10.1136/ard.2006.051672 PMC1798498

[B21] Cimaz R Coppa GV Kone-Paut I Joint contractures in the absence of inflammation may indicate mucopolysaccharidosis Pediatr Rheumatol Online J 2009 7 18 10.1186/1546-0096-7-18 19852785 PMC2775028

[B22] Tessitore A Pirozzi M Auricchio A Abnormal autophagy, ubiquitination, inflammation and apoptosis are dependent upon lysosomal storage and are useful biomarkers of mucopolysaccharidosis VI Pathogenetics 2009 2 4 doi:10.1186/1755-8417-2-4 10.1186/1755-8417-2-4 PMC2708151 19531206

[B23] Cho T-J Lehmann W Edgar C Tumor necrosis factor alpha activation of the apoptotic cascade in murine articular chondrocytes is associated with the induction of metalloproteinases and specific pro-resorptive factors Arthitis Rheum 2003 48 2845 2854 10.1002/art.11390 14558090

[B24] Machold KP Nell V Stamm T Aletaha D Smolen JS Early rheumatoid arthritis Curr Opin Rheutmatol 2006 18 282 288 10.1097/01.bor.0000218950.27483.80 16582693

[B25] Gabrielli O Clarke LA Bruni S Coppa GV Enzyme-replacement therapy in a 5-month-old boy with attenuated presymptomatic MPS I: 5-year follow-up Pediatrics 2010 125 e183 e187 10.1542/peds.2009-1728 20026495

[B26] McGill JJ Inwood AC Coman DJ Enzyme replacement therapy for mucopolysaccharidosis VI from 8 weeks of age-a sibling control study Clin Genet 2010 77 492 498 10.1111/j.1399-0004.2009.01324.x 19968667

[B27] Burrow TA Hopkin RJ Leslie ND Tinkle BT Grabowski GA Enzyme reconstitution/replacement therapy for lysosomal storage diseases Curr Opin Pediatr 2007 19 628 635 10.1097/MOP.0b013e3282f161f2 18025928

